# A Bayesian Belief Network for Murray Valley encephalitis virus risk assessment in Western Australia

**DOI:** 10.1186/s12942-016-0036-x

**Published:** 2016-01-28

**Authors:** Soon Hoe Ho, Peter Speldewinde, Angus Cook

**Affiliations:** School of Population Health (M431), The University of Western Australia, 35 Stirling Highway, Crawley, Perth, WA 6009 Australia; Centre of Excellence in Natural Resource Management, The University of Western Australia, Perth, WA Australia; The Albany Centre, 35 Stirling Terrace, Albany, WA 6332 Australia

**Keywords:** Murray Valley encephalitis virus, Bayesian Belief Network, Risk model, Risk maps, Western Australia

## Abstract

**Background:**

Murray Valley encephalitis virus (MVEV) is a clinically important virus in Australia responsible for a number of epidemics over the past century. Since there is no vaccine for MVEV, other preventive health measures to curtail its spread must be considered, including the development of predictive risk models and maps to help direct public health interventions. This article aims to support these approaches by presenting a model for assessing MVEV risk in Western Australia (WA).

**Methods:**

A Bayesian Belief Network (BBN) for assessing MVEV risk was developed and used to quantify and map disease risks in WA. The model combined various abiotic, biotic, and anthropogenic factors that might affect the risk of MVEV into a predictive framework, based on the ecology of the major mosquito vector and waterbird hosts of MVEV. It was further refined and tested using retrospective climate data from 4 years (2000, 2003, 2009, and 2011).

**Results:**

Implementing the model across WA demonstrated that it could predict locations of human MVEV infection and sentinel animal seroconversion in the 4 years tested with some degree of accuracy. In general, risks are highest in the State’s north and lower in the south. The model predicted that short-term climate change, based on the Intergovernmental Panel on Climate Change’s A1B emissions scenario, would decrease MVEV risks in summer and autumn, largely due to higher temperatures decreasing vector survival.

**Conclusions:**

To our knowledge, this is the first model to use a BBN to quantify MVEV risks in WA. The models and maps developed here may assist public health agencies in preparing for and managing Murray Valley encephalitis in the future. In its current form, the model is knowledge-driven and based on the analysis of potential risk factors that affect the dynamics of MVEV using retrospective data. Further work and additional testing should be carried out to test its validity in future years.

**Electronic supplementary material:**

The online version of this article (doi:10.1186/s12942-016-0036-x) contains supplementary material, which is available to authorized users.

## Background

At a global level, amongst the most important groups of emerging infectious diseases are those caused by arboviruses. These include West Nile virus (North America, 1999), Rift Valley Fever virus (Arabia, 2000), and Chikungunya virus (Indian Ocean rim, 2005/2006; Italy, 2007) [1, 2]. Many arboviral diseases are zoonoses maintained in a transmission cycle between a non-human vertebrate host and an arthropod vector [1].

In Australia, Murray Valley encephalitis virus (MVEV) is an important pathogenic arbovirus largely endemic to the Kimberley region of Western Australia (WA) and the Top End of the Northern Territory (NT) [3]. A number of MVE epidemics have occurred in the past century [3, 4], with the last major event in 1974, when 58 cases were reported across the country [4]. There are fears that with increased economic activity and development in northern Australia where the virus is endemic, the risk of MVEV epidemics in that region might increase, which could lead to its spread to, or emergence in, other parts of the country [5].

MVEV is maintained in an arthropod vector-vertebrate host transmission cycle. The virus’s major vector is the common banded mosquito, *Culex annulirostris* Skuse, a freshwater species, and the major hosts are waterbirds of the order *Ciconiiformes*, in particular the Rufous night heron, *Nycticorax caledonicus* [3]. Evidence also suggests that other species of mosquito may transmit the virus, such as *Cx. australicus*, *Aedes normanensis*, and *Ae. sagax* [6]. Although non-avian vertebrates such as kangaroos, rabbits, cattle, horses, pigs and mice can become infected with MVEV, their roles in transmitting the virus on to humans are uncertain [3, 7].

Cases of MVEV infection in humans are typically reported after the annual wet season in Australia, particularly during late summer and autumn [3]. In the majority of cases, infections are typically asymptomatic, and only about 0.10–0.67 % of all infected persons will display symptoms [8]. Although this is a small percentage, such infections can be lethal [8]. In about 40 % of symptomatic cases, permanent neurological sequelae may result [8]. Death can occur in about 15–30 % of encephalitic cases [8]. Currently, treatment of symptomatic infections is limited and no antiviral therapy has so far proven effective [8]. Preventive measures and education form the mainstay of public health efforts to control the virus.

As mentioned above, there is the prospect of increased incidence of MVEV infections due to greater human activity in northern Australia. For example, large parts of the Kimberley and Pilbara regions in WA are being developed to support mining, agriculture and other industries, and have driven increased immigration to those regions [5]. The increase in population and frequent travel-related exposure of those regions put communities there at greater risk of experiencing an outbreak. Compounding these processes are other factors such as climate change, which could possibly lead to changes in the geographical ranges of *Cx. annulirostris* or *Ciconiiformes*, and the emergence of MVEV in other parts of the country.

The factors that drive MVEV epidemics in the past have been identified and various models developed in an attempt to predict when and where the next outbreak will occur. According to Forbes, two preceding seasons of excessive rainfall is predictive of an MVEV epidemic in the Murray Valley region [9], while according to Nicholls, summer epidemics in that same region are most likely to occur if the Southern Oscillation Index is below average during the preceding three seasons [10]. Kay et al. (1987) developed a mathematical model of MVEV amplification specific to southern parts of Australia calibrated with data from the 1951 and 1974 epidemics [11], while Schuster et al. [12] devised a separate model to predict MVEV epidemics in the Kimberley and Pilbara regions of WA based on remotely sensed rainfall data. It predicted higher risk of MVEV with elevations in the monthly rainfall and the number of days with above average rainfall [12].

Here, we present a new approach for assessing MVEV risk in Western Australia. Our model differs from previous attempts by being a Bayesian Belief Network (BBN), incorporating a range of abiotic, biotic and anthropogenic factors that might affect features such as the population densities of *Ciconiiformes* and *Cx. annulirostris*, which would in turn affect MVEV risk. These include (i) climatic factors such as rainfall, temperature and humidity; (ii) geographical factors such as the presence of rivers and waterbodies; (iii) ecological factors that influence the timing of waterbird breeding and migration; and (iv) anthropogenic factors such as the seroprevalence of MVEV among members of the community. Risk maps encompassing all of Western Australia were then produced based on the model.

BBNs are acyclic graphical networks consisting of a set of vertices and edges (nodes and arrows, respectively) that represent conditional probability relationships between random variables, with each node having one or several states whose probabilities are assigned based on a prior distribution model (input or ‘parentless’ nodes) or calculated using Bayes’ Theorem from prior probabilities (‘child’ nodes) [13, 14]. BBNs are widely used in diverse fields such as artificial intelligence, medical diagnosis, speech recognition, and most relevantly, in ecology and environmental health as well [13–17]. For example, BBNs have been used in conjunction with Geographic Information Systems (GIS) to identify suitable habitats for wildlife [18, 19]; support conservation and land-management efforts [20]; evaluate forest management techniques [14]; analyse risk factors contributing to the outbreak of wildfires [21]; and also to assess environmental factors affecting the distribution of birth defects [22].

We chose a BBN as the modelling tool because it is best suited to modelling large and complex systems with multiple interacting variables [17], which is often the case in ecological processes including those that drive the emergence and distribution of MVEV. BBNs are generally robust to imperfect knowledge and approximate probabilities (even educated guesses) very often give good results [15, 23]. Because arboviruses are maintained in such complex ecological networks involving at least three different species—the viruses themselves, their vertebrate hosts, and arthropod vectors, each governed by its own ecological parameters and inhabiting its own niche in space and time—they are intrinsically well suited to risk modelling and mapping [24]. The factors that drive their emergence in new locations are complex and multifaceted, with landscape factors and ecological processes playing a dominant role. This extends to MVEV, whose emergence can only happen when viruses, vectors, hosts, and humans, are present in sufficient numbers simultaneously [1, 24].

The model that we present is an ‘expert system’ [23] designed after a comprehensive review of the literature. It was subsequently tested and refined using climatic data and historical reports during the main MVEV season of 4 years in the first decade of this century (2000, 2003, 2009 and 2011), containing a mix of epidemic and non-epidemic years. In such BBNs, where all or the majority of conditional probability tables (CPTs) are essentially determined by expert-opinion, there inevitably arises a sense of arbitrariness to the entire construction, although guidelines have been suggested by some authors to streamline and rationalise the whole process [16]. Here we present a unique way of populating expert-derived CPTs. As described further in the “Methods” and the Additional file 1, for every CPT that was to be populated by subjective opinion, we first assigned a numerical score/weight to every possible combination of parent node states. We then derived the probability distribution for that combination of states from a probability distribution table containing the distributions for all possible scores. These pre-defined probability distribution tables were carefully constructed to be symmetrically balanced around the middle score. The main advantage of using this method is that a consistent way of populating opinion-based CPTs was achieved.

The risk maps are presented in order to demonstrate the model’s capacity to predict MVEV outbreaks during the four selected years. Because rainfall has been consistently identified as a major factor affecting MVEV risk, we also included maps showing the risk distributions at different states of the rainfall node in order to test the model’s sensitivity to this particular node. Our results show significant differences in risk distributions across WA between ‘high’ and ‘low’ states of rainfall.

Finally, it is our hope that the model and maps presented here will add to the range of surveillance measures available to combat this infectious disease in Western Australia.

## Results

### Risk model

**Fig. 1:**
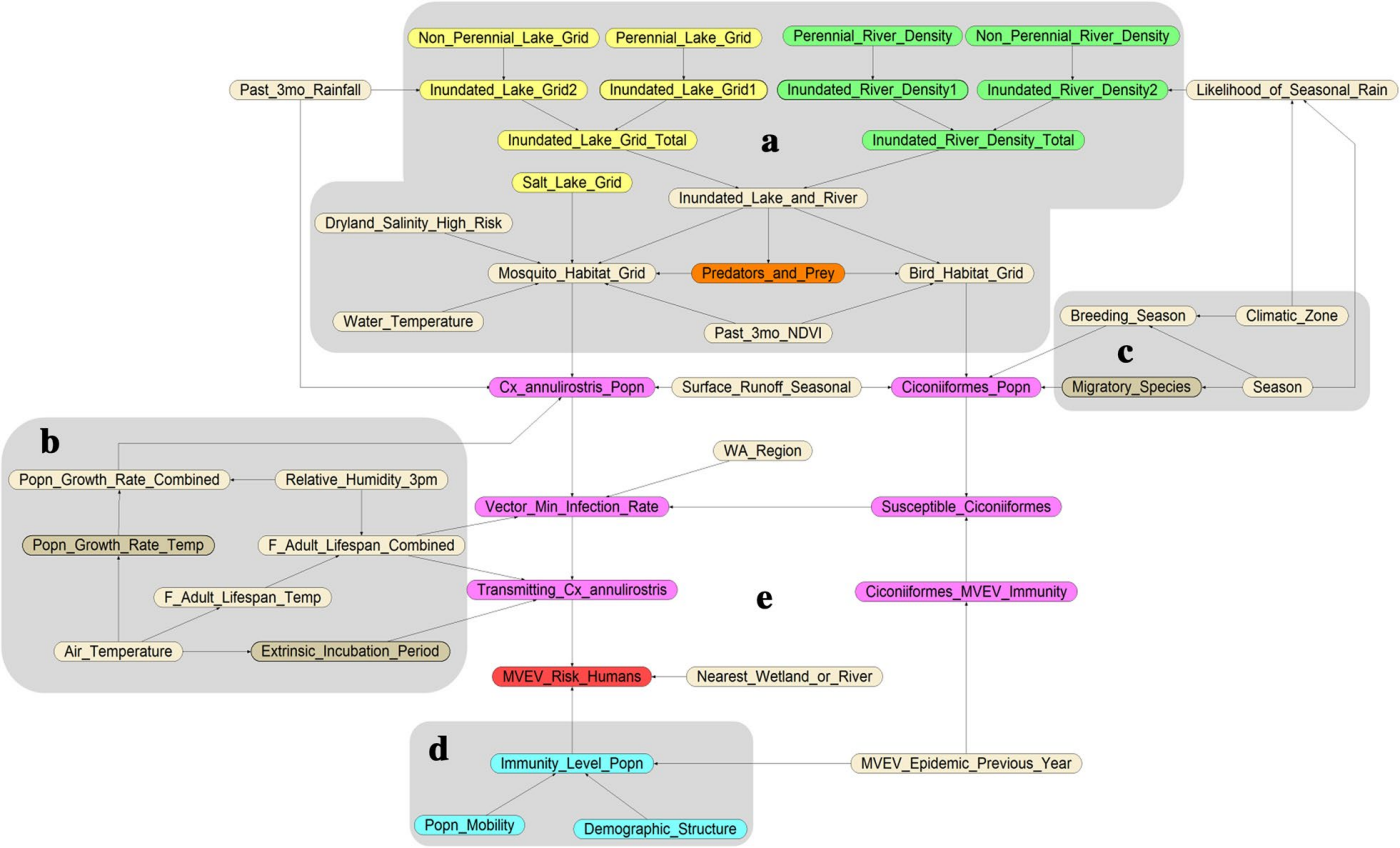
The infectious disease risk model for MVEV

The MVEV risk model is shown in Fig. 1. All node states and prior distributions are listed in Table 1, and the Conditional Probability Tables (CPTs) are provided in the Additional file 1. Prior distributions of parentless nodes are uniform while those of all other nodes are determined by their CPTs.

**Table 1:** Nodes and states of the MVEV BBN

Type of variable	Description of node; name in bold italics; node states; prior distribution (%)
Binary (2 states)	1. Is the location at high risk of dryland salinity (***Dryland_Salinity_High_Risk***) i.e. Description of node (***Node name***)
	Yes (50.00) i.e. state 1 (probability)
	No (50.00) i.e. state 2 (probability)
	2. Vegetation cover over the previous 3 months (***Past_3mo_NDVI***)
	No_vegetation (50.00)
	Vegetation_present (50.00)
	3. What is the likelihood of seasonal rainfall (***Likelihood_of_Seasonal_Rain***)
	High (50.00)
	Low (50.00)
	4. What is the seasonal amount of surface runoff (***Surface_Runoff_Seasonal***)
	Zero (50.00)
	Above_zero (50.00)
	5. Is it the breeding season for waterbirds (Breeding_Season)
	Yes (45.80)
	No (54.20)
	6. What is the direction of migratory movement of waterbirds (***Migratory_Species***)
	Arrival (50.00)
	Departure (50.00)
	7. Was there an MVEV epidemic last year (***MVEV_Epidemic_Previous_Year***)
	Yes (50.00)
	No (50.00)
	8. Linear distance to nearest waterbody (***Nearest_Wetland_or_River***)
	Below_15 km (50.00)
	Equal_or_Above_15 km (50.00)
Nominal/ordinal scale (3 states)	1. Density of mosquito predators and waterbird prey (***Predators_and_Prey***)
	High (43.40)
	Medium (49.60)
	Low (6.99)
	2. In which climatic zone is the location (***Climatic_Zone***)
	Tropical_WA (33.30)
	Arid_WA (33.30)
	Temperate_WA (33.30)
	3. Endemicity of MVEV in WA, according to the region (***WA_Region***)
	Kimberley (33.30)
	Pilbara (33.30)
	Rest_of_WA (33.30)
	4. Prevalence of *Ciconiiformes’s* immunity to MVEV (***Ciconiiformes_MVEV_Immunity***)
	High (25.00)
	Medium (50.00)
	Low (25.00)
	5. Proportion of new immigrants in the human community (***Popn_Mobility***)
	High (3.30)
	Medium (33.30)
	Low (33.30)
	6. Proportion of under-15 year olds in the human community (***Demographic_Structure***)
	Higher_Propn_Young (33.30)
	Medium_Propn_Young (33.30)
	Lower_Propn_Young (33.30)
	7. Prevalence of immunity to MVEV in the human community (***Immunity_Level_Popn***)
	High (30.30)
	Medium (39.40)
	Low (30.30)
	8. Overall risk of MVEV at the location (***MVEV_Risk_Level***)
	High (2.24)
	Medium (5.54)
	Low (92.20)
Nominal/ordinal scale (4 states)	1. Abundance of non-perennial lakes at the location ***(Non_Perennial_Lake_Grid)***
	High (25.00)
	Medium (25.00)
	Low (25.00)
	Zero (25.00)
	2. Abundance of perennial lakes at the location (***Perennial_Lake_Grid***)
	High (25.00)
	Medium (25.00)
	Low (25.00)
	Zero (25.00)
	3. Density of non-perennial rivers at the location (***Non_Perennial_River_Density***)
	High (25.00)
	Medium (25.00)
	Low (25.00)
	Zero (25.00)
	4. Density of perennial rivers at the location (***Perennial_River_Density***)
	High (25.00)
	Medium (25.00)
	Low (25.00)
	Zero (25.00)
	5. Level of inundation of the perennial lakes at the location (***Inundated_Lake_Grid1***)
	High (25.00)
	Medium (25.00)
	Low (25.00)
	Zero (25.00)
	6. Level of inundation of the non-perennial lakes at the location (***Inundated_Lake_Grid2***)
	High (18.80)
	Medium (22.90)
	Low (27.10)
	Zero (31.30)
	7. Level of inundation of the perennial rivers at the location (***Inundated_River_Density1***)
	High (25.00)
	Medium (25.00)
	Low (25.00)
	Zero (25.00)
	8. Level of inundation of the non-perennial rivers at the location (***Inundated_River_Density2***)
	High (12.50)
	Medium (21.90)
	Low (28.10)
	Zero (37.50)
	9. Level of inundation of all lakes at the location (***Inundated_Lake_Grid_Total***)
	High (48.20)
	Medium (26.00)
	Low (18.00)
	Zero (7.81)
	10. Level of inundation of all rivers at the location (***Inundated_River_Density_Total***)
	High (43.40)
	Medium (27.30)
	Low (19.90)
	Zero (9.38)
	11. Level of inundation of all lakes and rivers at the location (***Inundated_Lake_and_River***)
	High (79.30)
	Medium (15.00)
	Low (5.03)
	Zero (0.73)
	12. Abundance of saline lakes at the location (***Salt_Lake_Grid***)
	High (25.00)
	Medium (25.00)
	Low (25.00)
	Zero (25.00)
	13. What is the season of the year (***Season***)
	Spring (25.00)
	Summer (25.00)
	Autumn (25.00)
	Winter (25.00)
	14. What is the effect of air temperature on the *Cx. annulirostris* growth rate (***Popn_Growth_Rate_Temp***)
	Ideal (11.10)
	Positive_fast (22.20)
	Positive_slow (22.20)
	Negative (44.40)
	15. What is the combined effect of air temperature and relative humidity on the *Cx. annulirostris* growth rate (***Popn_Growth_Rate_Combined***)
	Ideal (14.80)
	Positive (21.80)
	Neutral (19.00)
	Negative (44.40)
Nominal/ordinal scale (5 states)	1. How suitable is the location as a habitat for *Cx. annulirostris* (***Mosquito_Habitat_Grid***)
	Very_Good (4.96)
	Good (10.10)
	Neutral (11.10)
	Poor (5.86)
	Very_Poor (67.90)
	2. How suitable is the location as a habitat for *Ciconiiformes* (***Bird_Habitat_Grid***)
	Very_Good (35.10)
	Good (34.60)
	Neutral (21.20)
	Poor (7.12)
	Very_Poor (2.01)
	3. What is the population density of *Cx. annulirostris* at the location (***Cx_annulirostris_Popn***)
	Very_High (1.02)
	High (3.44)
	Medium (5.91)
	Low (5.63)
	Very_Low (84.00)
	4. What is the population density of *Ciconiiformes* at the location (***Ciconiiformes_Popn***)
	Very_High (20.90)
	High (33.40)
	Medium (26.90)
	Low (13.80)
	Very_Low (4.88)
	5. What is the combined effect of air temperature and relative humidity on female adult *Cx. annulirostris*’ longevity (F_Adult_Lifespan_Combined)
	Very_long (11.50)
	Long (16.80)
	Average (18.80)
	Short (13.10)
	Very_short (6.54)
	Unsuitable_conditions (33.30) [*this state is only invoked if the state of* ***F_Adult_Lifespan_Temp*** = *Unsuitable_Temp. See* ***Table S11***.]
	6. Minimum MVEV infection rate of *Cx. annulirostris* at the location (***Vector_Min_Infection_Rate***)
	Very_High (0.33)
	High (1.81)
	Medium (4.54)
	Low (6.56)
	Very_Low (86.80)
	7. Population density of MVEV-transmitting *Cx. annulirostris* at the location (***Transmitting_Cx_annulirostris***)
	Very_High (0.14)
	High (1.04)
	Medium (3.29)
	Low (6.17)
	Very_Low (89.30)
	8. Population density of *Ciconiiformes* susceptible to MVEV at the location (***Susceptible_Ciconiiformes***)
	Very_High (11.50)
	High (27.40)
	Medium (29.60)
	Low (21.10)
	Very_Low (10.40)
Categories based on numerical ranges	1. Total rainfall over the previous 3 months (***Past_3mo_Rainfall***)
	Below_60 mm (33.30)
	From_60 mm_to_100 mm (33.30)
	Above_100 mm (33.30)
	2. Surface temperature of waterbodies over the previous 3 months (***Water_Temperature***)
	From_20C_to_30C (33.30)
	Below_10C_or_Above_40C (33.30)
	All_other_Temp (33.30)
	3. Average air temperature over the previous 3 months (***Air_Temperature***)
	Below_18C (11.10)
	From_18_to_21C (11.10)
	From_21_to_24C (11.10)
	From_24_to_27C (11.10)
	From_27_to_30C (11.10)
	From_30_to_33C (11.10)
	From_33_to_36C (11.10)
	From_36_to_39C (11.10)
	Above_39C (11.10)
	4. Average relative humidity at 3 pm over the previous 3 months (***Relative_Humidity_3*** ***pm***)
	From_0_to_30 (33.33)
	From_30_to_60 (33.33)
	From_60_to_100 (33.33)
	5. MVEV’s extrinsic incubation period in *Cx. annulirostris* (Extrinsic_Incubation_Period)
	From_0_to_5_days (11.10)
	From_5_to_10_days (22.20)
	From_10_to_15_days (22.20)
	From_15_to_20_days (11.10)
	Unsuitable_Temp (33.30)
	6. Effect of average air temperature on female adult *Cx. annulirostris*’ lifespan (***F_Adult_Lifespan_Temp***)
	From_25_to_30_days (11.10)
	From_20_to_25_days (25.90)
	From_15_to_20_days (7.41)
	From_10_to_15_days (7.41)
	From_5_to_10_days (7.41)
	From_0_to_5_days (7.41)
	Unsuitable_Temp (33.30)

Names of every node are in bold italics and bracketed; every node state is listed with its probability bracketed

The rationale behind the construction of the BBN, including reasons for the inclusion of all variables, is provided in the Additional file 1. Briefly, in determining the risk of MVEV, the model takes four main factors into account: population density of *Cx. annulirostris* (titled **Cx_annulirostris_Popn**); population density of *Ciconiiformes* (titled **Ciconiiformes_Popn**); endemicity of the virus by region within WA (titled **WA_Region**); and immune status of the human community (titled **Immunity_Level_Popn**). In turn, the population densities of mosquito vectors and vertebrate hosts are affected by the habitat suitability of the area (Part A), and seasonal climatic factors that affect the population densities of *Cx. annulirostris* (Part B) and *Ciconiiformes* (Part C). Age distribution and migrant background of the human community are two factors that might also affect the immune status of the community, with younger individuals and new migrants (assuming they had arrived from non-MVEV endemic regions) being more susceptible to infection since they were presumably less exposed to the virus in the past, thereby increasing the community’s risk (Part D).

The first three nodes mentioned above converge on the node which models the minimum infection rate of *Cx. annulirostris* with MVEV (titled **Vector_Min_Infection_Rate**). This variable is dependent on the lifespan of adult females since older adults are expected to have experienced a longer period of potential exposure to the virus than younger adults and so are more likely to have higher infection rates [Lindsay, pers. comm.]. This node directly affects the density of virus-transmitting vectors, which is itself dependent on the longevity of adult female mosquitoes and the extrinsic incubation period of the virus in *Cx. annulirostris*. Long-lived adult vectors increase the transmission rate by taking multiple blood meals from hosts [Lindsay, pers. comm.]. The extrinsic incubation period (EIP) is inversely proportional to transmission rate as shorter EIP means shorter time taken between infection of the vector and onward transmission.

The query node (titled **MVEV_Risk_Humans**) has three states corresponding to ‘low’, ‘medium’ and ‘high’ risks. It is affected by the density of transmitting *Cx. annulirostris*; overall susceptibility of the human community to infection; and distance to the nearest lake or river (which reflects the fact that *Cx. annulirostris* are more likely to be found within 15 km of wetlands due to their short flight range [25]).

### Risk maps

#### Average current climatic conditions

**Fig. 2:**
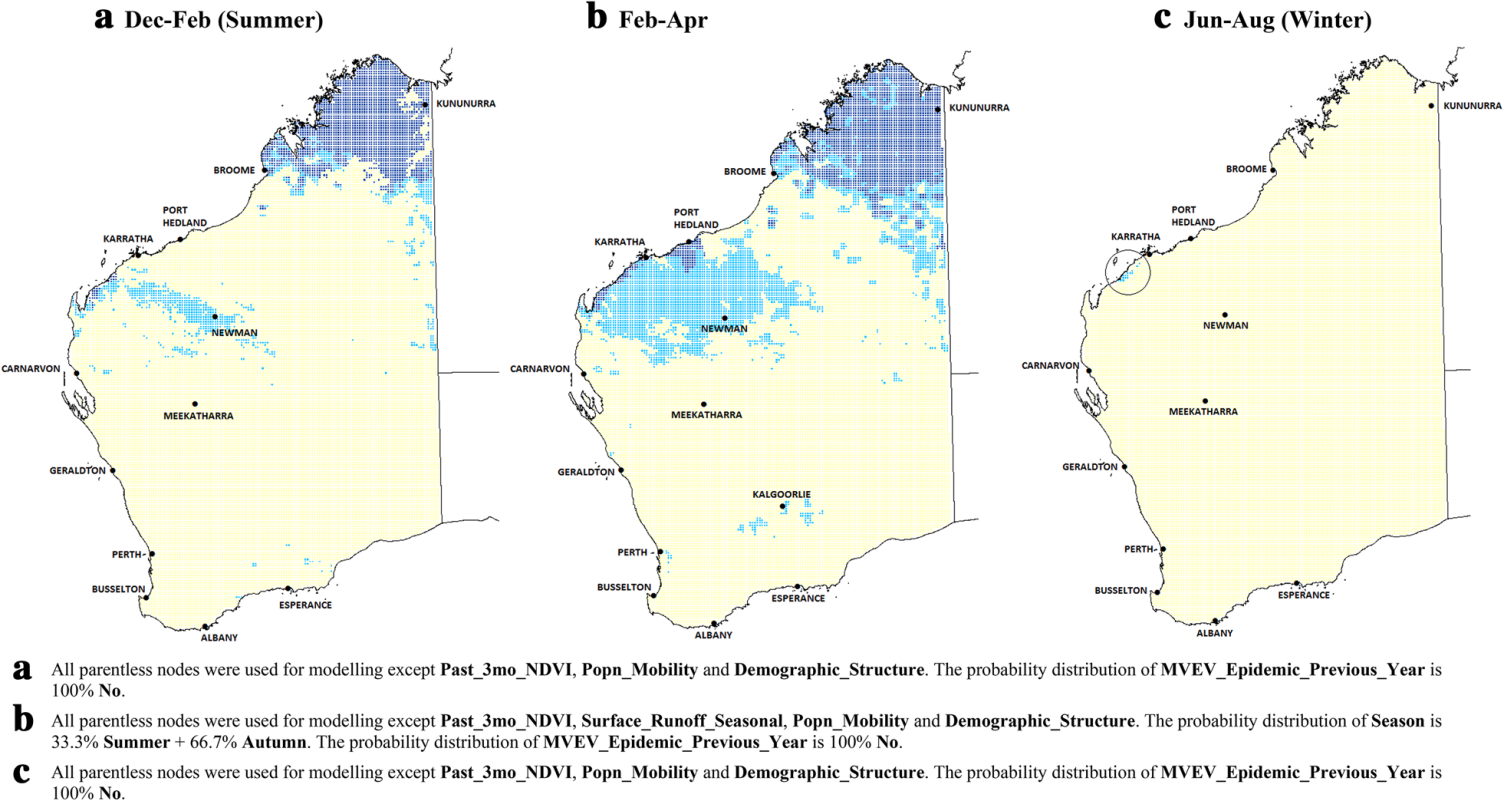
Average current climatic conditions. MVEV risk maps under current average climatic conditions, December–February (summer) (**a**), February–April (**b**), and June–August (winter) (**c**) (*dark blue* ‘**High**’; *light blue* ‘**Medium**’; *rest* ‘**Low**’ risk)

MVEV risk across Australia was modelled under current average climatic conditions (Fig. 2). The time period with highest risk is from February to April. The model correctly predicted that the Kimberley region is most at risk of MVEV: it is in the ‘**High**’ risk category from December to April (Fig. 2a, b). In the Pilbara, an area in the ‘**Medium**’ risk category runs along a narrow corridor from Newman to the coast in a northwest-southeast direction in **summer** (Fig. 2a), **but** expands to include a substantially larger area in the succeeding three month period (Fig. 2b). It is during this later period that the major towns of Karratha and Port Hedland are included within the ‘**High**’ risk zone.

Apart from the Kimberley and Pilbara regions, the rest of WA mostly has ‘**Low**’ risk. Interestingly, Perth and the area around Kalgoorlie have ‘**Medium**’ risk from February to April (Fig. 2b), largely due to a greater abundance of lakes and rivers there. Three-monthly rainfall is sufficient to cause these waterbodies to become inundated with water thus providing breeding sites for mosquitoes.

In winter (Fig. 2c), the total area with ‘**Medium**’ or ‘**High**’ risk contracts to include only the region west of Karratha, in line with expectations since the winter climate does not support large populations of mosquito vectors.

#### Scenario modelling: current climatic conditions with maximum rainfall

**Fig. 3:**
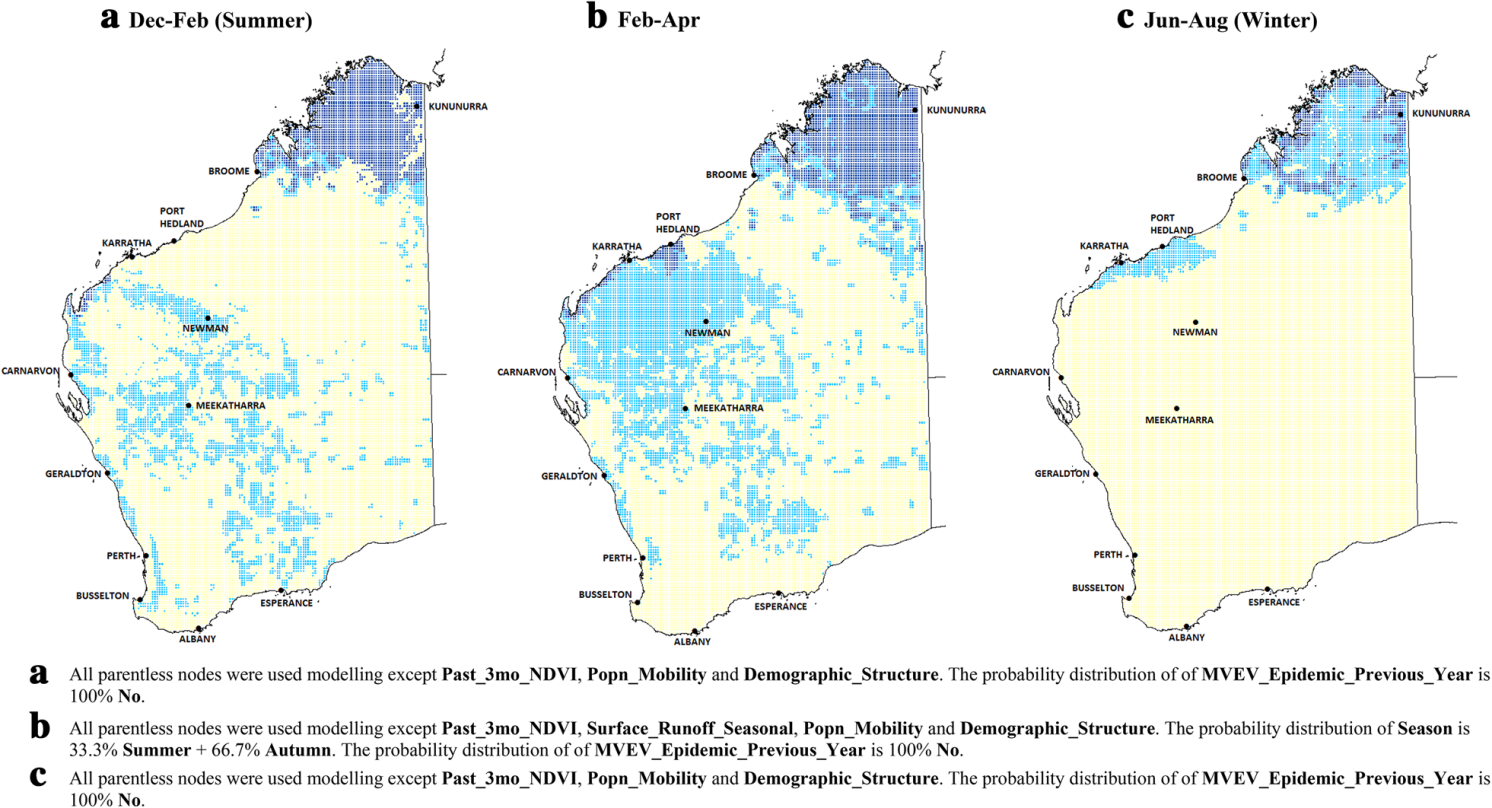
Scenario modelling: current climatic conditions with maximum rainfall. MVEV risk maps with elevated rainfall patterns (above 100 mm over the 3 month periods) throughout Western Australia, but having current average temperature and relative humidity (*dark blue* ‘**High**’; *light blue* ‘**Medium**’; *rest* ‘**Low**’ risk)

Figure 3 illustrates the risk distributions if every part of WA were to experience the highest three-monthly rainfall state (in excess of 100 mm) in all three time periods, while maintaining the current average values for temperature and relative humidity. While this is an unlikely situation, higher levels of rainfall can occur in parts of WA when there are cyclones or due to the La Nina pattern [26].

Larger areas of WA have ‘**Medium**’ or ‘**High**’ risk, extending as far south as Albany and Esperance during summer (Fig. 3a). These places are near natural wetlands and rivers and are expected to support large populations of *Cx. annulirostris* and *Ciconiiformes* if rainfall is high. Inland towns such as Meekatharra are also at higher risk from December to April (Fig. 3a, b). MVEV transmission could even occur in the Kimberley region during winter if the seasonal rainfall experienced is greater than 100 mm (Fig. 3c), although that does not normally occur [27].

#### Scenario modelling: current climatic conditions with minimum rainfall

**Fig. 4:**
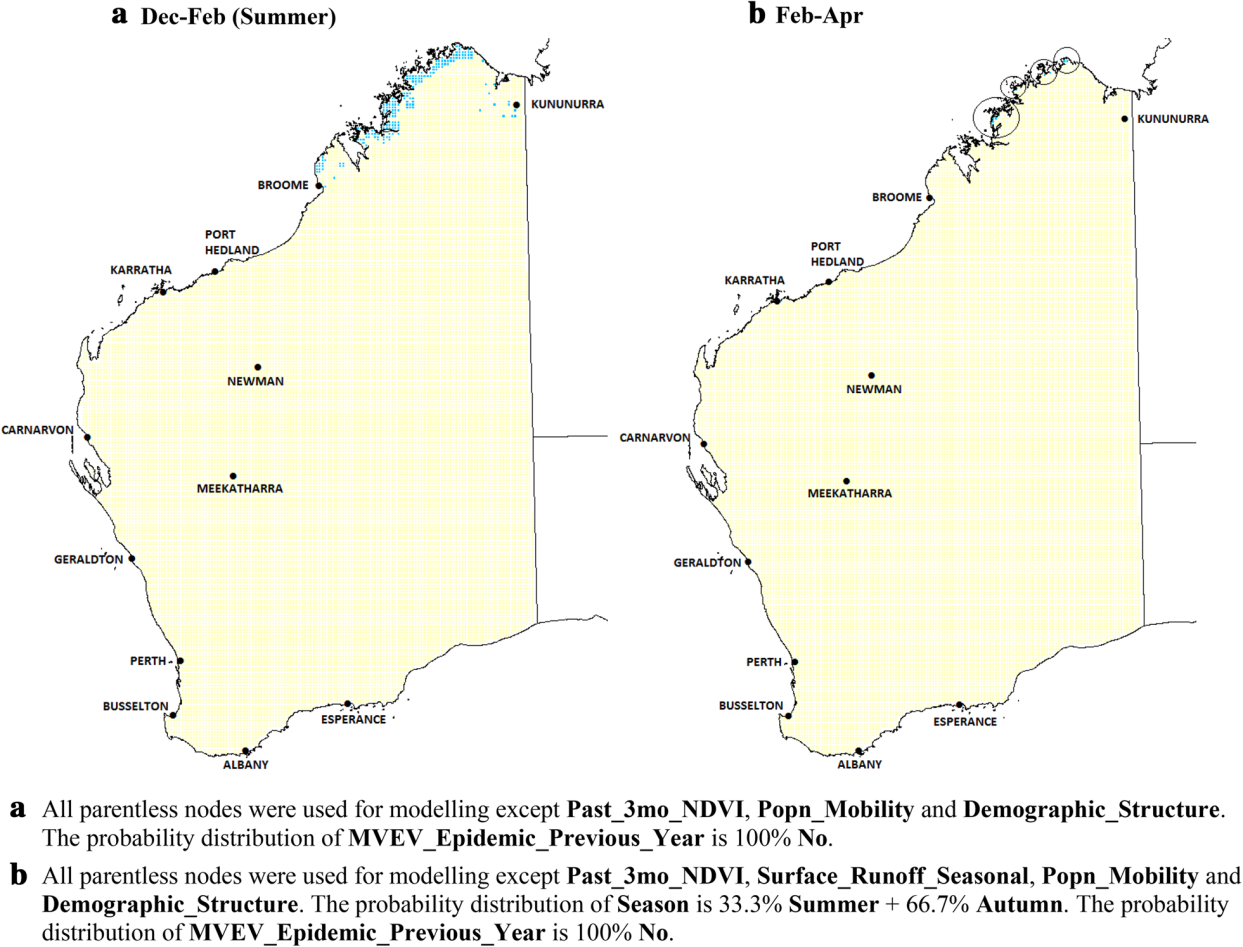
Scenario modelling: current climatic conditions with minimum rainfall. MVEV risk maps with low rainfall levels (less than 60 mm over the 3 month periods) throughout Western Australia, but having current average temperature and relative humidity. During winter, the risk is ‘**Low**’ throughout WA. (*light blue* ‘**Medium**’; *rest* ‘**Low**’ risk)

Figure 4 shows the risk distributions if the total three-monthly rainfall were to fall below 60 mm throughout WA (e.g. during drought conditions), while maintaining current average values for temperature and relative humidity. In this case, no location on the map is at ‘**High**’ risk. Areas at ‘**Medium**’ risk include a narrow band in the extreme north along the coast during summer plus the region around Kununurra (Fig. 4a). From February to April, the total area at ‘**Medium**’ risk shrinks even further, this time excluding Kununurra (Fig. 4b). In winter, the entire State is expected to have ‘**Low**’ risk.

Figures 3 and 4 illustrate the importance of rainfall in determining MVEV risk level, in agreement with a number of other studies that also demonstrate the importance of this variable [12, 28, 29].

#### Case study: February–April, 2000

**Fig. 5:**
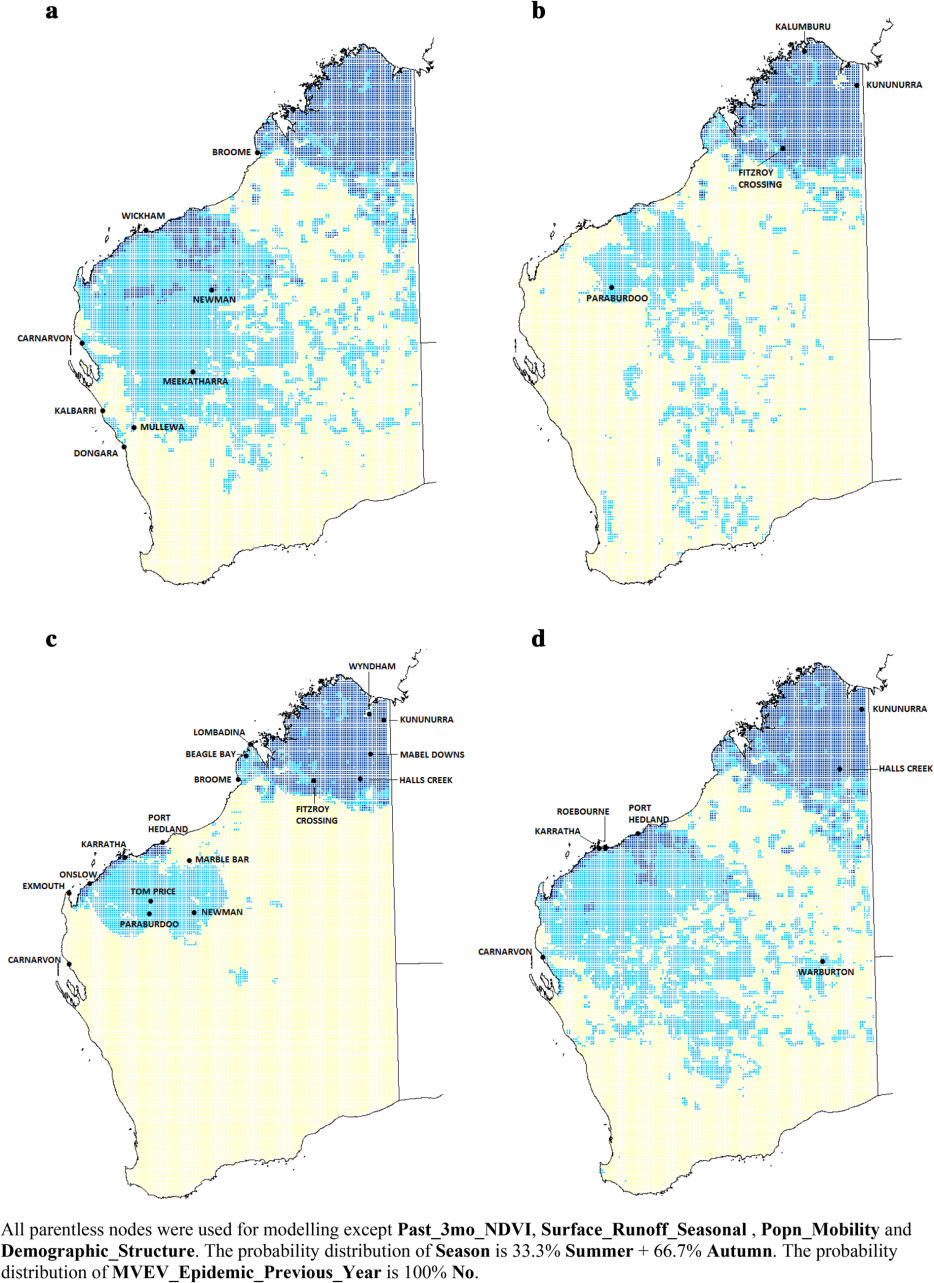
Case study: February–April 2000 (**a**), case study: February–April 2003 (**b**), case study: February–April 2009 (**c**) and case study: February–April 2011 (**d**) (*dark blue* ‘**High**’; *light blue* ‘**Medium**’, *rest* ‘**Low**’ risk)

Figure 5a shows the predicted MVEV risk distribution in WA using actual climate data from February to April 2000. During the summer and autumn of 1999/2000, WA experienced unusually high amounts of rainfall. This pattern was compounded by Tropical Cyclone Steve which moved along the entire coastal region of northern WA from Kununurra to Shark Bay before turning inland and moving southeasterly towards Esperance [30, 31].

With significant parts of the State experiencing elevated levels of rainfall, the model predicted that larger areas should be at ‘**Medium**’ or ‘**High**’ risk of MVEV transmission. In addition to the usual locations in the north (which continue to have ‘**High**’ risk of transmission), the model suggested that more southerly areas are now at ‘**Medium**’ risk, including large parts of the Midwest (which should normally have a ‘**Low**’ risk category during this period; see Fig. 2b).

The model’s predictions of affected areas were checked against historical reports. During that season, nine cases of MVEV infection in WA were recorded [30]. They were Newman (6 March), Kalbarri (8 April), Carnarvon (16 April), Meekatharra (20 April), an unknown location in the Midwest to Kimberley region (25 April), Wickham (28 April), Mullewa (3 May), Dongara (5 May), and Broome (8 May). Comparing these places with areas marked ‘**Medium**’ or ‘**High**’ risk in Fig. 5a reveals that they are all included within ‘**Medium**’ to ‘**High**’ risk zones. The last three cases had onset of symptoms in early May but the dates suggest that they had probably acquired their infections in late April/early May.

#### Case study: February–April, 2003

Figure 5b shows the predicted MVEV risk distribution using actual climate data from February to April 2003. Year 2003 was chosen to determine how the model performed for a non-epidemic year. The total rainfall in the summer/autumn of 2002/2003 was much less than that in 2000, although the Kimberley region still had above average levels of rain [32].

Once again, the Kimberley region is predicted to have the highest risk of MVEV transmission from February to April, but the total area in the Pilbara having ‘**Medium**’ to ‘**High**’ risk is smaller than average (see Fig. 2b) and much smaller than the total area during the same period in 2000. Interestingly, the model predicted that ‘**Medium**’ risk areas should extend further south than usual all the way to Esperance on the southern coast.

There was no notified human case of MVEV infection in 2003, and we therefore compared the predicted risk distribution with sentinel chicken serosurvey results. That season was associated with little flavivirus activity throughout Australia [33]. In WA, MVEV was first detected in February at Fitzroy Crossing, followed by seroconversions at Kununurra, Kalumburu and Paraburdoo in April and May. No further seroconversions were reported for the rest of that season [33]. These four sites are all situated within what the model predicted were ‘**High**’ or ‘**Medium**’ risk areas. Southern/inland regions of WA (in a vertical band from the central Pilbara region down to Esperance) had zones predicted to be at ‘**Medium**’ MVEV risk. However, it was difficult to correlate these estimates with actual measures of MVEV activity because sentinel chicken flocks were not stationed at these regions (in that year, the southernmost town where a flock was stationed was York, about 100 km east of Perth [34]).

#### Case study: February–April, 2009

We tested the model with climatic data from February to April 2009 (Fig. 5c). The total area where the risk is ‘**Medium**’ or ‘**High**’ is more restricted in 2009 compared to the same period in 2000 and 2003, but appears similar to the modelled situation under current average climatic conditions (see Fig. 2b).

Four cases of human MVEV infection were reported across Australia in 2009 with two from WA: one at Broome in March and another at Port Hedland in May [35]. Those two centres were correctly predicted as falling within or near to ‘**High**’ risk zones. Additionally, data from sentinel chicken flocks indicated that MVEV activity was greater during the 2009 season than in previous years, with seroconversions occurring in flocks at Kununurra, Halls Creek, Sally Malay Mine (Mabel Downs), Fitzroy Crossing, Harding Dam, and “all locations where sentinel chickens were in place in the Kimberley, Pilbara and Gascoyne regions” [35]. A number of those locations are indicated to fall within ‘**Medium**’ or ‘**High**’ risk areas. The model also correctly predicted that “no seroconversions to MVEV were detected south of the Gascoyne region” [35], labelling those regions as having ‘**Low**’ risk.

#### Case study: February–April, 2011

The model was tested using climate data from February to April 2011 (Fig. 5d). Year 2011 was another major epidemic year with 17 cases reported across Australia, including three deaths [36]. Nine cases were from WA including one death [36]. Predicted ‘**High**’ risk areas include much of the Kimberley and coastal areas of the Pilbara region, while ‘**Medium**’ risk areas extend as far south as Kalgoorlie.

A comparison of Fig. 5d with a map of actual MVEV cases in 2011 [36] shows a high degree of agreement between the two. There was even an unusual case occurring far inland near the border with Northern Territory and South Australia (actual location unknown). The large area having ‘**Medium**’ to ‘**High**’ risk is attributable to greater amounts of rainfall, which led to large populations of *Cx. annulirostris* and *Ciconiiformes*, similar to the situation which existed in February to April 2000. The 2011 experience showed that MVEV activity is not restricted to coastal regions: cases can occur within WA’s interior, as modelled by the BBN.

#### Future scenario: risk in 2030

**Fig. 6:**
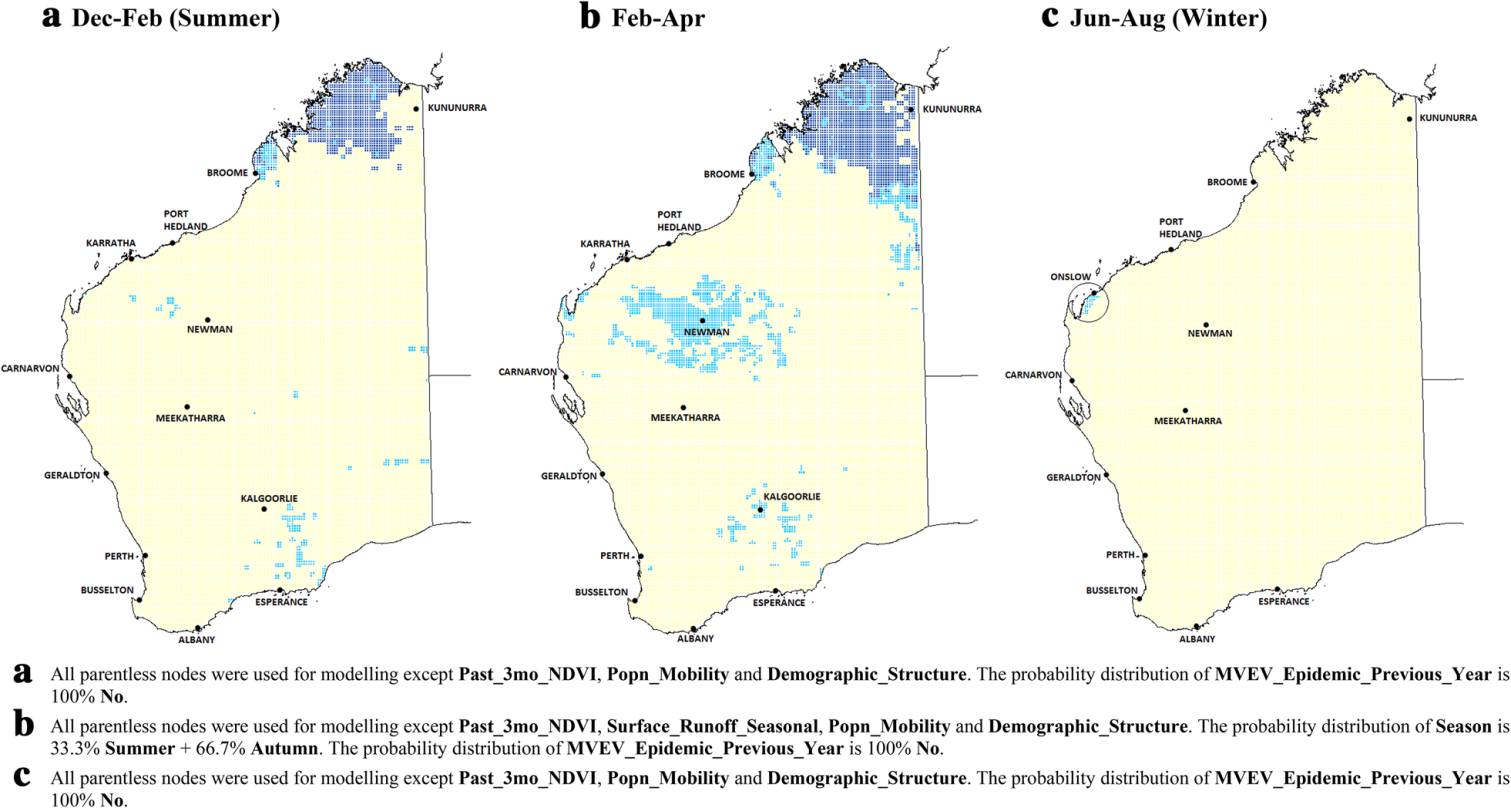
Future scenario: risk in 2030. Predicted MVEV risk throughout WA in three periods of 2030, according to the A1B SRES emissions scenario [37], with climate predictions generated using the CSIRO-Mk3.5 model under a moderate rate of global warming (climate prediction software: OzClim [38]) (*dark blue* ‘**High**’; *light blue* ‘**Medium**’; *rest* ‘**Low**’ risk)

Finally, we ran the model using predicted climate data for the year 2030 (Fig. 6), under the SRES A1B emissions scenario which is based on a future world condition of high economic growth, a global population that is assumed to peak in 2050, and a balanced use of world energy sources between fossil and non-fossil fuels [37]. Climate predictions were generated under the CSIRO Mark 3.5 (CSIRO-Mk3.5) model by OzClim [38].

Comparing Figs. 2 and 6, the overall MVEV risk is in fact predicted to decline between now and 2030. From December to April 2030, the total area having ‘**Medium**’ to ‘**High**’ risk is smaller compared to the current situation. Coastal areas of the Pilbara region no longer have higher risks of MVEV transmission. During winter, however, the risk distribution is predicted to be almost the same as the current situation.

#### Sensitivity analysis of the query node

Sensitivity analysis of a BBN node is a procedure that allows users to quantify the amount of influence every other node has on that node [16]. For a node with discrete states, it is based on the reduction in the ‘entropy’ of that node when a finding has been entered for one of the other nodes [16]. Sensitivity analysis of the node for MVEV risk in humans (titled **MVEV_Risk_Humans**) showed that it was particularly affected by the following five nodes:

1. Density of MVEV-transmitting *Cx. annulirostris* (**Transmitting_Cx_annulirostris**)
2. *Cx. annulirostris*’ minimum MVEV infection rate (**Vector_Min_Infection_Rate**)
3. *Ciconiiformes*’ population density (**Cx_annulirostris_Popn**)
4. Habitat suitability for *Cx. annulirostris* (**Mosquito_Habitat_Grid**)
5. High risk of dryland salinity (**Dryland_Salinity_High_Risk**)

The five most important abiotic factors affecting MVEV risk are, in order:

1. High risk of dryland salinity (**Dryland_Salinity_High_Risk**)
2. Average air temperature (**Air_Temperature**)
3. Surface water temperature of waterbodies (**Water_Temperature**)
4. Linear distance to nearest waterbody (**Nearest_Wetland_or_River**)
5. Abundance of saline lakes (**Salt_Lake_Grid**)

Rainfall is not within the top five most influential nodes. However, this does not mean that it is not an important factor affecting MVEV risk, because the sensitivity of a node to the state of another is acutely affected by the number of intermediate nodes separating them, in addition to other factors such as the CPTs [16]. Although **Past_3mo_rainfall** is not included amongst the five most important abiotic factors affecting **MVEV_Risk_Humans**, its influence is exerted through its effects on other nodes such as **Cx_annulirostris_Popn**. The strength of this influence is seen in Figs. 3 and 4.

## Discussion

In this paper, we present a novel Bayesian Belief Network-based model for assessing Murray Valley encephalitis virus (MVEV) risk in Western Australia. Although this application of BBNs to MVEV risk modelling has not previously been developed, the use of such networks has been successfully applied in other aspects of ecological modelling (e.g. [14, 18, 19]). Compared with other modelling techniques, BBNs have the distinct advantage of being able to incorporate expert-derived knowledge and uncertainty in an explicit manner, allowing users to test the consequences of different suggested possibilities (i.e. hypotheses) on the outcome [23]. Naturally, this gives rise to the possibility of bias since different experts may weigh the importance of causative factors differently. In addition, since the networks cannot handle continuous variables, these must be discretised first, introducing another aspect of subjectivity [16, 23]. However, these drawbacks are reduced by the fact that the network and every assumption is made fully explicit, allowing for easy evaluation and critique, and more importantly, for easy updating and refinement as well [16, 23].

The MVEV risk model presented in this article is one such ‘expert-derived’ system, based on the ‘nidus’ concept of disease transmission originally proposed by Pavloskiy (cited in [39]). In the context of arboviruses, it states that disease transmission can only take place when three components—the virus, competent vectors and susceptible hosts—are simultaneously present at a particular location. Susceptible humans will only get infected when they enter the transmission zone or nidus [39].

The MVEV transmission nidus was modelled by the parent nodes of the *Cx. annulirostris* minimum MVEV infection rate node (titled **Vector_Min_Infection_Rate**). The entire BBN revolves around them and all other nodes in the network can be thought of as ‘secondary’ nodes supporting these ‘primary’ nodes: the node for *Cx. annulirostris* population density (titled **Cx_annulirostris_Popn**) models the density of infected and uninfected vectors at that location; the node for susceptible *Ciconiiformes* population density (titled **Susceptible_Ciconiiformes**) does the same for the vertebrate hosts of MVEV; the node for MVEV endemicity by region (titled **WA_Region**) approximates the size of the virus population there by reference to where the virus is enzootic or epizootic in WA (assuming that virus populations are highest on average in enzootic regions and lowest in places that are neither enzootic nor epizootic).

Generally, regions most conducive for creating MVEV transmission nidi are those surrounding lakes (wetlands) and rivers. Therefore on a State-wide scale across WA, MVEV risk is primarily determined by the proximity to lakes (wetlands) and rivers, which are the main habitats of both *Cx. annulirostris* and *Ciconiiformes*. The risk maps show that for an average year (i.e. in which climatic parameters have their current mean values), locations south of 25^o^S (the latitude of the coastal town of Carnarvon) are generally not at risk of experiencing an MVEV outbreak. North of this latitude, MVEV risk is highest from February to April, where total rainfall is highest of the three time periods considered. In winter, the total area at risk shrinks until only the region west of Karratha can support MVEV outbreaks throughout the year. This region has an abundance of wetlands to support large waterbird populations [40] and has a suitable climate all year round for mosquito breeding.

The model shows that rainfall patterns had a large impact on MVEV activity in WA. The obvious explanation for this is that rainfall directly impacts the population densities of *Cx. annulirostris* and *Ciconiiformes* in an area. For example, during the two major epidemic years (2000 and 2011), northern WA experienced summer cyclones which brought large quantities of rain over much of that region [41]. The importance of rainfall on the level of risk also brings the model in line with a number of other studies [e.g. 12, 28, 29].

Interestingly, MVEV risk was shown to decline for the 2030 scenario, compared to the current situation. That is because of predicted higher mean temperatures from summer to April of 2030 over large parts of WA. Temperatures above 33 °C are not ideal for *Cx. annulirostris* growth [42], and this led to the lowering of MVEV transmission risk in 2030.

The overall agreement between the predictions and historical reality increases our confidence in the ability of the model to accurately predict future MVEV risks in WA. However, note that during the 4 years studied, places where human MVEV cases and sentinel chicken seroconversions occurred were only a small fraction of the total area predicted to have average to above average (‘**Medium**’ or ‘**High**’) risk. This shows that the model may have adequate sensitivity but still has relatively poor specificity, and this could continue to be refined in future BBNs.

There are other potential limitations in this analysis. The overall influence of vegetation on mosquito and waterbird habitat suitability was kept low (Tables S6 and S7 in the Additional file 1). This was partly because vegetation was classified very broadly in terms of ‘presence’ or ‘absence’. In addition, risk here was modelled on a large State-wide scale and thus a highly detailed picture of the vegetative landscape, including its effects on the abundance of *Cx. annulirostris* and *Ciconiiformes*, was not necessary. It is likely to become more important when a model is devised to assess MVEV risk at smaller, more localised areas. That would require careful analysis of the types of vegetation present [43, 44].

Human-related nodes in the model were not used during the mapping process. These nodes incorporate the idea that the risk of MVEV in a community is inversely related to the level of immunity in that community. Their effects need to be considered when comparing MVEV risks between different communities in WA, but were not used in the mapping process because the scale of the risk map to be produced is too small to represent such differences clearly. The population of WA is highly clumped, with the majority living in urban areas. On a State-wide level, these urban areas appear simply as dots on a landscape surrounded by vast tracts of unpopulated land.

This points to a wider limitation of the analysis in terms of spatial resolution, since the resolution of the risk maps is limited by the resolution of the input data. For example, the resolution of the nodes representing waterbody and river densities is approximately 600 km^2^ (see “Methods”), which represents the smallest resolution of the risk maps. We would like to emphasise that the maps are meant to delineate risk distributions on a small scale covering all 2.5 million km^2^ of land within WA. While the resolution is too coarse to allow for greater precision in locating potential outbreak areas, the fact that cases of MVEV infection and seroconversion coincide with ‘high’ and ‘medium’ risk regions on the risk maps is an indication that the principles behind the model are valid.

Furthermore, risks were modelled in three-month blocks, and all climatic data were averaged throughout that timeframe before being used as inputs for the model. Thus the BBN can only model general relationships between climate and vector and virus population growth characteristics. This is suitable when dealing with broad trends in disease risk across very large landmasses, which is the scale for which the MVEV model in this study was intended. Microclimatic variations in climate variables were not accounted for; these will become important when developing models that deal with risk on a micro-scale (e.g. a few suburbs of Perth).

## Conclusion

MVEV is a significant pathogen affecting public health in Western Australia and is expected to remain so for the foreseeable future. This study presents a Bayesian Belief Network (BBN)-based risk model for MVEV risk assessment in WA, built on considerations of vector and host ecologies and designed for application on a State-wide scale. In its current form, the MVEV risk model is knowledge-driven and based on an analysis of potential risk factors that might affect the dynamics of this disease. The results and future applications of the BBN could potentially assist health authorities with outbreak prediction and the allocation of resources to combat MVEV in the future.

## Methods

### Framework of BBN risk modelling and mapping

The modelling software was Netica™ (Norsys Software Corp., Vancouver, BC) while mapping was performed on ArcGIS v10.1 (Esri, Redlands, CA).

A comprehensive review of the literature was conducted to determine the main factors affecting the distribution of MVEV in Australia and Western Australia specifically. An initial BBN was created and GIS-compatible spatial data of environmental factors were sourced from government agencies (Table 2). Risk distributions were modelled in the following 3-month blocks: December–February (summer); February–April (peak MVEV season); and June–August (winter) to assess seasonal effects on risk [45], and all climatic data were averaged throughout these three-month periods.

**Table 2:** Spatial data and sources used to generate the MVEV risk map

Spatial data	Source map (data type)	Agency (release date)	References
Geographical features			
Lakes (perennial and non-perennial)	Global Map Australia 1M 2001 and Geodata Topo 2.5 M 2003 (Esri Polygon Shapefile)	National Mapping Division, Geoscience Australia (July 2004)	[46, 47]
Saline lakes	Present Vegetation—Post European Settlement (1988) (Esri Polygon Shapefile)	National Mapping Division, Geoscience Australia (July 2004)	[48]
Rivers (perennial and non-perennial)	Global Map Australia 1M 2001 (Esri Line Shapefile)	National Mapping Division, Geoscience Australia (July 2004)	[46]
WA regions	Local Government Areas ASGS Non ABS Structures Ed 2012 Digital Boundaries in ESRI Shapefile Format (Esri Polygon Shapefile)	Australian Bureau of Statistics (July 2012)	[54]
Environmental features			
Seasonal surface runoff	Surface runoff map; in Primary Data: Water data for use with MCAS-S Version 3 software (Esri Raster Image)	Multi-Criteria Analysis Shell for Spatial (MCAS-S) Decision Support Version 3: DATA—2011; ABARES, Department of Agriculture (October 2011)	[52]
Dryland salinity	Western Australia Dryland Salinity Risk Assessment 2000 (Esri Polygon Shapefile)	Australia Dryland Salinity Assessment Spatial Data (1:2,500,000)—NLWRA 2001; ABARES, Department of Agriculture (Date not provided)	[51]
Climatic features			
Mean air temperature	Average seasonal and monthly maximum, minimum, and mean temperature (1961–1990), and Maps of recent and past conditions (ASCII Grid File)	Climate Data Online, Bureau of Meteorology, Australia (Current)	[27, 32]
Total rainfall	Average seasonal and monthly rainfall (1961–1990), and Maps of recent and past conditions (ASCII Grid File)	Climate Data Online, Bureau of Meteorology, Australia (Current)	[27, 32]
Mean 3 pm relative Humidity	Average seasonal and monthly 3 pm relative humidity (1976–2005), and Maps of recent and past conditions (ASCII Grid File)	Climate Data Online, Bureau of Meteorology, Australia (Current)	[27, 32]
Climatic zone	Agroclimatic Zone map, in Overlays (contextual) for use with MCAS-S Version 3 software (Esri Polygon Shapefile)	Multi-Criteria Analysis Shell for Spatial (MCAS-S) Decision Support Version 3: DATA—2011; ABARES, Department of Agriculture (October 2011)	[52]

Risk maps were generated based on the initial BBN and assessed to determine whether they conformed to expectations based on the literature. Revisions were made where necessary before the draft model and maps were consulted with an external professional (Dr. Michael Lindsay, Department of Health of Western Australia); any suggestions provided were subsequently factored in and the BBN revised again. The model was further refined using retrospective climate data from 4 years (2000, 2003, 2009 and 2011) to determine whether it could accurately predict locations where human infections and/or sentinel chicken seroconversions occurred. The entire process was knowledge-driven, iterative, and continued until a risk model and risk maps were obtained that conformed to expectations and retrospective data from the literature.

### Bayesian Belief Network model-building

All variables were represented as nature nodes with discrete states, and arrows were inserted between nodes that formed a causal pathway. Conditional Probability Tables (CPTs) were populated using data from the literature, where available. Where data were unavailable, the CPTs were populated according to the following procedure: (i) the degree of influence of every parent node state on the child node was estimated and weighted with a numerical score, before the total weight of every combination of parent node states was obtained by summing the scores; (ii) the summed scores of every combination of parent node states were re-scaled to a 100-point scale using a standardised approach; and (iii) the corresponding probability distributions of the child node were then read off or interpolated.

Classification schemes for all nodes are detailed further in the Additional file 1. For nodes where we have greater freedom to choose the total number of states, we selected classification schemes where a ‘median value’ state exists. The two most economical schemes where a median value exists are those having three- or five-states in total, corresponding to ‘high/middle/low’, and ‘very high/high/middle/low/very low’ states. In some nodes such as **Perennial_Lake_Grid** where there are four states, the fourth state corresponds to the ‘zero value’, hence allowing one of the other three states to be the ‘median value’ state. This method of classification only applies to nodes where we have a measure of freedom to select the number of states; they are usually populated by the ‘point-system’ mentioned above. Nodes such as **F_Adult_Lifespan_Temp** and **Air_Temperature,** which have seven and nine states respectively, are not affected because their states were not subjectively determined, i.e. they correspond to objective measures such as lifespan and temperature.

### Input data processing and classification

All input nodes were used during the modelling and mapping process except the human factor nodes and the node for vegetation presence (titled **Past_3mo_NDVI**). The node **Surface_Runoff_Seasonal** was used for risk modelling and mapping in summer and winter only, because accurate data for February to April were unavailable. The vegetation node was not used for mapping partly because vegetation was classified very broadly in terms of ‘presence’ or ‘absence’, and partly because risk was modelled on a large State-wide scale hence a highly detailed picture of the vegetative landscape and its effects on the abundance of *Cx. annulirostris* and *Ciconiiformes* was not necessary. Human factor nodes were not used because the population distribution in WA is highly aggregated; their effects are not noticeable on small-scale maps as in this analysis.

#### Non-saline lakes (perennial and non-perennial) and saline lakes

A rectangular ‘fishnet’ grid composed of 160 × 160 cells was created, spanning 112°–156° west to east and −9° to −40° north to south. Each cell measured 0.275° × 0.19375°, equating to approximately 600 km^2^. ‘Inland water’ features were obtained from ‘Global Map Australia 1M 2001’ [46]. Features whose hydrological category was ‘Perennial/Permanent’ were classified as ‘perennial lakes’, while those that were either ‘Non-Perennial/Intermittent/Fluctuating’ or ‘Unknown’ were classified as ‘non-perennial lakes’. ‘Swamp’ features from ‘Geodata Topo 2.5 M 2003’ [47] were also included in the dataset for ‘perennial lakes’. Saline lakes (identified from ‘Present Vegetation—Post European Settlement (1988)’ [48]) were excluded and analysed separately.

Lake features were overlaid with the fishnet grid. The percentage of each grid cell occupied by ‘perennial lakes’ and ‘non-perennial lakes’ was calculated. The two percentage lists were combined and summary statistics (excluding the value 0 %) obtained. Grid cells were then re-classified as follows: **Zero**: 0 %; **Low**: 0–33rd percentile; **Medium**: 33rd–67th percentile; **High**: 67th–100th percentile. A similar method was used to process saline lakes features.

#### Rivers (perennial and non-perennial)

‘Watercourse’ line features were obtained from [46]. Features whose hydrological category was ‘Perennial/Permanent’ were classified as ‘perennial rivers’ while those that were ‘Non-Perennial/Intermittent/Fluctuating’ were classified as ‘non-perennial rivers’. ‘Aqueduct/canal’ features from [46] were also included in the dataset for ‘perennial rivers’. Kernel densities of ‘perennial rivers’ and ‘non-perennial rivers’ lines were calculated with a search radius twice that of output cell size. Raw density values of the two datasets were combined and summary statistics (excluding the value 0 unit per square map units) obtained. Grid cells were re-classified using the Zero/Low/Medium/High categories as for ‘Lakes’ above.

#### Climatic parameters

Monthly and seasonal temperature, rainfall, and 3 pm relative humidity ASCII grid files were obtained from the Australian Bureau of Meteorology [27, 32]. Air temperature was used as a surrogate for water temperature, a reasonable approximation at the water surface where *Cx. annulirostris*’ eggs, larvae and pupae are present, but not at lower depths (temperature of stream water is correlated with (although not strictly equal to) air temperature [49]). This assumption excludes the effects of other factors that might affect surface water temperature, such as shading from vegetation and the type of substrate [50].

#### Dryland salinity

Spatial data for dryland salinity was obtained from the ‘Western Australia Dryland Salinity Risk Assessment 2000’ [51]. Area polygons classified as having ‘high’ risk of dryland salinity in the year 2000 were selected and overlaid with the fishnet grid, and cells that enclosed those polygons were re-classified **Yes** (i.e. high risk of dryland salinity), while the rest were re-classified **No**.

#### Seasonal surface runoff

Mean summer and winter surface runoff maps were obtained from the ‘Multi-Criteria Analysis Shell for Spatial (MCAS-S) Decision Support Version 3.1’ [52]. The maps were re-classified as follows: **Zero**: where runoff = 0 ML; or **Above_Zero**: where runoff > 0 ML.

#### Climatic zones of WA

A map of WA agroclimatic zones was obtained from [53] and re-classified according to tropical, arid, and temperate regions.

## Additional file

10.1186/s12942-016-0036-x Full description of the MVEV risk model.

## Abbreviations

BBN: Bayesian Belief Network
GIS: Geographic Information System
MVE: Murray Valley encephalitis
MVEV: Murray Valley encephalitis virus
WA: Western Australia
