# Supplementary material for: A Bayesian Belief Network for Murray Valley encephalitis virus risk assessment in Western Australia
Source: Int J Health Geogr. 2016 Jan 28;15:6. doi: 10.1186/s12942-016-0036-x (PMC4730662; doi:10.1186/s12942-016-0036-x)
Supplement: Supplementary file 1 — 10.1186/s12942-016-0036-x Full description of the MVEV risk model. [file 12942_2016_36_MOESM1_ESM.docx]

**Detailed description of the MVEV risk model**

**Vector and host habitat requirements (Part A)**

This part models the main natural habitats of *Cx. annulirostris* and *Ciconiiformes* (i.e. places where natural bodies of surface water are present. Areas with high densities of lakes (wetlands) and rivers are highly conducive for both vector and hosts to survive and thrive, so the risk of MVEV is generally higher in those places [3, 53]. These areas delineate very generally the geographical extent of high MVEV risk areas.

**Table S1:** The CPTs of **Inundated_Wetland_Grid1** (left) and **Inundated_River_Density1** (right).

| **Perennial_**  **Wetland_Grid** | **Inundated_**  **Wetland_Grid1** |  |  | **Perennial_**  **River_Density** | **Inundated_**  **River_Density1** |
| --- | --- | --- | --- | --- | --- |
| **High** | High |  |  | **High** | High |
| **Medium** | Medium |  |  | **Medium** | Medium |
| **Low** | Low |  |  | **Low** | Low |
| **Zero** | Zero |  |  | **Zero** | Zero |

These tables were populated based on the assumption that perennial waterbodies are constantly inundated with water throughout the year.

**Table S2:** The CPT of **Inundated_Lake_Grid2**.

| **Non_Perennial_Lake_Grid** | **Past_3mo_Rainfall** | **Inundated_Lake_Grid2** | | | |
| --- | --- | --- | --- | --- | --- |
|  |  | **High** | **Medium** | **Low** | **Zero** |
| **High** | **Below_60mm** | 25 | 50 | 25 | 0 |
| **High** | **From_60mm_to_100mm** | 100 | 0 | 0 | 0 |
| **High** | **Above_100mm** | 100 | 0 | 0 | 0 |
| **Medium** | **Below_60mm** | 0 | 25 | 50 | 25 |
| **Medium** | **From_60mm_to_100mm** | 0 | 100 | 0 | 0 |
| **Medium** | **Above_100mm** | 0 | 100 | 0 | 0 |
| **Low** | **Below_60mm** | 0 | 0 | 50 | 50 |
| **Low** | **From_60mm_to_100mm** | 0 | 0 | 100 | 0 |
| **Low** | **Above_100mm** | 0 | 0 | 100 | 0 |
| **Zero** | **Below_60mm** | 0 | 0 | 0 | 100 |
| **Zero** | **From_60mm_to_100mm** | 0 | 0 | 0 | 100 |
| **Zero** | **Above_100mm** | 0 | 0 | 0 | 100 |

This table is based on empirical data showing that temporary wetlands in arid Australia are inundated when the total rainfall over the preceding three months exceeds 60mm [54]; hence a 1:1 correspondence between the parent and child nodes exists when that occurs. A total three-monthly rainfall of 100mm was chosen as the cut-off value for the highest state, following [29]. The probability distributions in other rows were assigned based on consensus-based estimates by the research team.

**Table S3:** The CPT of **Inundated_River_Density2**.

| **Non_Perennial_River_Density** | **Likelihood_of_Seasonal_Rain** | **Inundated_River_Density2** | | | |
| --- | --- | --- | --- | --- | --- |
|  |  | **High** | **Medium** | **Low** | **Zero** |
| **High** | **High** | 75 | 25 | 0 | 0 |
| **High** | **Low** | 25 | 50 | 25 | 0 |
| **Medium** | **High** | 0 | 75 | 25 | 0 |
| **Medium** | **Low** | 0 | 25 | 50 | 25 |
| **Low** | **High** | 0 | 0 | 75 | 25 |
| **Low** | **Low** | 0 | 0 | 50 | 50 |
| **Zero** | **High** | 0 | 0 | 0 | 100 |
| **Zero** | **Low** | 0 | 0 | 0 | 100 |

These probability distributions were assigned based on consensus-based estimates by the research team. Intuitively, non-perennial rivers are more likely to be inundated during the wet season, so when **Likelihood_of_Seasonal_Rain** = **High**, the probability distribution of **Inundated_River_Density2** is skewed towards a 1:1 relationship with **Non_Perennial_River_Density**. When **Likelihood_of_Seasonal_Rain** = **Low**, the most probable state of **Inundated_River_Density2** was set at one level lower than the state of **Non_Perennial_River_Density**.

**Table S4:** The CPTs of **Inundated_Lake_Grid_Total**, **Inundated_River_Density_Total** and **Inundated_Lake_and_River**.

| **Inundated_Lake_Grid1**  ***Or***  **Inundated_River_Density1**  ***Or***  **Inundated_Lake_Grid_Total** | **Inundated_Lake_Grid2**  ***Or***  **Inundated_River_Density2**  ***Or***  **Inundated_River_Density_Total** | **Inundated_Lake_Grid_Total**  ***Or***  **Inundated_River_Density_Total**  ***Or***  **Inundated_Lake_and_River** | | | |
| --- | --- | --- | --- | --- | --- |
|  |  | **High** | **Medium** | **Low** | **Zero** |
| **High** | **High** | 100 | 0 | 0 | 0 |
| **High** | **Medium** | 100 | 0 | 0 | 0 |
| **High** | **Low** | 100 | 0 | 0 | 0 |
| **High** | **Zero** | 100 | 0 | 0 | 0 |
| **Medium** | **High** | 100 | 0 | 0 | 0 |
| **Medium** | **Medium** | 50 | 50 | 0 | 0 |
| **Medium** | **Low** | 50 | 50 | 0 | 0 |
| **Medium** | **Zero** | 0 | 100 | 0 | 0 |
| **Low** | **High** | 100 | 0 | 0 | 0 |
| **Low** | **Medium** | 50 | 50 | 0 | 0 |
| **Low** | **Low** | 0 | 50 | 50 | 0 |
| **Low** | **Zero** | 0 | 0 | 100 | 0 |
| **Zero** | **High** | 100 | 0 | 0 | 0 |
| **Zero** | **Medium** | 0 | 100 | 0 | 0 |
| **Zero** | **Low** | 0 | 0 | 100 | 0 |
| **Zero** | **Zero** | 0 | 0 | 0 | 100 |

The child nodes can be thought of as being equal to the ‘sum’ of the two parent nodes. As long as the state of one parent node is **High**, the state of the child node is also **High**. The rest of the table was populated based on consensus-based estimates by the research team.

**Table S5:** The CPT of **Predators_and_Prey**, depicting a general relationship between abundance of waterbodies and the abundance of predators and prey.

| **Inundated_Wetland_and_River** | **Predators_and_Prey** | | |
| --- | --- | --- | --- |
|  | **High** | **Medium** | **Low** |
| **High** | 50 | 50 | 0 |
| **Medium** | 25 | 50 | 25 |
| **Low** | 0 | 50 | 50 |
| **Zero** | 0 | 0 | 100 |

This table was populated based on consensus-based estimates by the research team. The population densities of *Cx. annulirostris’* predators and *Ciconiiformes*’ prey are expected to be positively correlated with the abundance of waterbodies in an area.

**Table S6** explains how the CPT for **Mosquito_Habitat_Grid** was populated using the ‘points’ system (see **Methods**). This system was used to populate a number of other CPTs as well, such as **Bird_Habitat_Grid**, **Cx_annulirostris_Popn** and **Popn_Growth_Rate_Combined**. All points were assigned based on our estimates of the effects that individual parent node states have on the child node.

**Table S6:** Points of parent node states for **Mosquito_Habitat_Grid** CPT.

| **Salt_Lake**  **_Grid** | | **Past_3mo_NDVI** | | **Water_Temperature** | | **Predators_**  **and_Prey** | | **Inundated_**  **Wetland_and**  **_River** | | **Dryland_Salinity_High_Risk** | |
| --- | --- | --- | --- | --- | --- | --- | --- | --- | --- | --- | --- |
| **High** | 0 | **No_Vegetation** | 0 | **From_20C_to_30C** | 6 | **High** | 0 | **High** | 6 | **Yes** | * |
| **Medium** | 2 | **Vegetation**  **_Present** | 2 | **All_other_temp** | 0 | **Medium** | 1 | **Medium** | 4 | **No** | 0 |
| **Low** | 4 |  | | **Below_10C_or**  **_Above_40C** | * | **Low** | 2 | **Low** | 2 |  | |
| **Zero** | 6 |  | | | | | | **Zero** | 0 |  |  |
| *For these states, **Mosquito_Habitat_Grid** = 100% **Very_Poor** regardless of the states of all other parent nodes.  *Illustrative Calculation (double underlined states):*  *Score = 2+2+0+1+4+0 = 9*  *Max. score = 6+2+6+2+6 = 22*  *Scaled score = 9/22*100 = 40.9091*  *The probability distribution of* ***Mosquito_Habitat_Grid*** *states is then obtained by interpolation from a pre-defined table (see* ***Methods****).*  *(N.B.: It is not the absolute magnitude of the points that is of concern. Rather, it is the size of the points relative to other parent nodes that is important.)* | | | | | | | | | | | |

*Cx. annulirostris* is salt-intolerant species, so regions with greater abundance of saline lakes or prone to dryland salinity are expected to have less of them. Vegetation provides shelter to mosquito larvae [44]. Survival of mosquito larvae was shown to be greatest when the water temperature is between 20^o^C and 30^o^C; temperatures below 10^o^C or above 40^o^C are lethal [42].

The node **Bird_Habitat_Grid** summarises the broad biotic and abiotic requirements that make a location suitable for *Ciconiiformes* to survive. *Ciconiiformes* are not intolerant of salt water, and they are not restricted by water temperature to the same extent as *Cx. annulirostris*. In their case, both **Predators_and_Prey** and **Past_3mo_NDVI** have a positive influence on habitat suitability. The effect of inter-specific competition was not considered since it is difficult to model accurately. The CPT of this node was populated using the ‘points system’ (**Table S7**). All points were assigned based on our estimates of the impact that the parent nodes and states they represent could have on **Bird_Habitat_Grid**.

**Table S7:** Points of parent node states for **Bird_Habitat_Grid** CPT.

| **Predators_and_Prey** | | **Past_3mo_NDVI (†)** | | **Inundated_Wetland_and_River** | |
| --- | --- | --- | --- | --- | --- |
| **High** | 6 | **No_Vegetation** | 0 | **High** | 6 |
| **Medium** | 3 | **Vegetation_Present** | 2 | **Medium** | 4 |
| **Low** | 0 |  | | **Low** | 2 |
|  | |  | | **Zero** | 0 |
| *Illustrative Calculation (double underlined states):*  *Score = 6+0+4 = 10*  *Max. score = 6+2+6 = 14*  *Scaled score = 10/14*100 = 71.429*  *Probability distribution of* ***Bird_Habitat_Grid*** *states is interpolated from a pre-defined table (see* ***Methods)****.* | | | | | |

†Vegetation provides breeding sites for colonial waterbirds [43].

**Factors affecting *Cx. annulirostris* population growth (Part B)**

**Table S8:** The CPT of **Popn_Growth_Rate_Temp**.

| **Air_Temperature** | **Popn_Growth_Rate_Temp** |
| --- | --- |
| **Below_18C** | Negative |
| **From_18_to_21C** | Positive_Slow |
| **From_21_to_24C** | Positive_Fast |
| **From_24_to_27C** | Ideal |
| **From_27_to_30C** | Positive_Fast |
| **From_30_to_33C** | Positive_Slow |
| **From_33_to_36C** | Negative |
| **From_36_to_39C** | Negative |
| **Above_39C** | Negative |

This CPT was populated based on the empirical determination of the effects of temperature on *Cx. annulirostris*’s population intrinsic rate of increase in [42].

**Table S9:** Points of parent node states of **Popn_Growth_Rate_Combined** CPT.

| **Popn_Growth_Rate_Temp** | | **Rel_Humidity_3pm (†)** | |
| --- | --- | --- | --- |
| **Ideal** | 2 | **0_to_30** | 0 |
| **Positive_Fast** | 1 | **30_to_60** | 1 |
| **Positive_Slow** | 0 | **60_to_100** | 2 |
| **Negative** | * |  | |
| *For this state, **Popn_Growth_Rate_Combined** is 100% **Negative** regardless of the state of the other parent node. | | | |

†Low relative humidity is lethal to mosquitoes [55]; cut-off values of 60% and 30% were selected (by our judgement) to differentiate between high, medium and low relative humidity.

**Table S10:** The CPT of **F_Adult_Lifespan_Temp**.

| **Air_Temperature** | **F_Adult_Lifespan_Temp** | | | | | | |
| --- | --- | --- | --- | --- | --- | --- | --- |
|  | **From_25_**  **to_30_days** | **From_20_**  **to_25_days** | **From_15_**  **to_20_days** | **From_10_**  **to_15_days** | **From_5_**  **to_10_days** | **From_0_**  **to_5_days** | **Unsuitable_Temp** |
| **Below_18C** | 0 | 0 | 0 | 0 | 0 | 0 | 100 |
| **From_18_to_21C** | 0 | 100 | 0 | 0 | 0 | 0 | 0 |
| **From_21_to_24C** | 66.667 | 33.333 | 0 | 0 | 0 | 0 | 0 |
| **From_24_to_27C** | 33.333 | 66.667 | 0 | 0 | 0 | 0 | 0 |
| **From_27_to_30C** | 0 | 33.333 | 66.667 | 0 | 0 | 0 | 0 |
| **From_30_to_33C** | 0 | 0 | 0 | 66.667 | 33.333 | 0 | 0 |
| **From_33_to_36C** | 0 | 0 | 0 | 0 | 33.333 | 66.667 | 0 |
| **From_36_to_39C** | 0 | 0 | 0 | 0 | 0 | 0 | 100 |
| **Above_39C** | 0 | 0 | 0 | 0 | 0 | 0 | 100 |

This CPT was populated based on empirical data from [42].

**Table S11:** Points of parent node states of **F_Adult_Lifespan_Combined** CPT.

| **F_Adult_Lifespan_Temp** | | **Rel_Humidity_3pm** | |
| --- | --- | --- | --- |
| **From_25_to_30_days** | 10 | **0_to_30** | 0 |
| **From_20_to_25_days** | 8 | **30_to_60** | 5 |
| **From_15_to_20_days** | 6 | **60_to_100** | 10 |
| **From_10_to_15_days** | 4 |  | |
| **From_5_to_10_days** | 2 |  |  |
| **From_0_to_5_days** | 0 |  |  |
| **Unsuitable_Temp** | * |  |  |
| *For this state, **F_Adult_Lifespan_Combined** = 100% **Unsuitable_Conditions** regardless of the state of the other parent node. | | | |

This CPT was populated based on our judgement of the effects of the two parent nodes: a decrease in relative humidity lowers the combined lifespan of *Cx. annulirostris*. Similarly, as the state of **F_Adult_Lifespan_Temp** decreases, the combined lifespan of *Cx. annulirostris* drops.

**Table S12:** The CPT of **Extrinsic_Incubation_Period**. The CPT restricts EIP (and MVEV transmission) to temperatures between 18 and 36^o^C.

| **Air_Temperature** | **Extrinsic_Incubation_Period** |
| --- | --- |
| **Below_18C** | Unsuitable_Temp |
| **From_18_to_21C** | From_15_to_20_days |
| **From_21_to_24C** | From_10_to_15_days |
| **From_24_to_27C** | From_10_to_15_days |
| **From_27_to_30C** | From_5_to_10_days |
| **From_30_to_33C** | From_5_to_10_days |
| **From_33_to_36C** | From_0_to_5_days |
| **From_36_to_39C** | Unsuitable_Temp |
| **Above_39C** | Unsuitable_Temp |

This table was populated using empirical data obtained by [56].

**Factors affecting *Ciconiiformes* population density (Part C)**

**Table S13:** The CPT of **Breeding_Season**.

| **Climatic_Zone** | **Season** | **Breeding_Season** | |
| --- | --- | --- | --- |
|  |  | **Yes** | **No** |
| **Tropical_WA** | **Spring** | 0 | 100 |
| **Tropical_WA** | **Summer** | 100 | 0 |
| **Tropical_WA** | **Autumn** | 50 | 50 |
| **Tropical_WA** | **Winter** | 0 | 100 |
| **Arid_WA** | **Spring** | 50 | 50 |
| **Arid_WA** | **Summer** | 50 | 50 |
| **Arid_WA** | **Autumn** | 50 | 50 |
| **Arid_WA** | **Winter** | 50 | 50 |
| **Temperate_WA** | **Spring** | 100 | 0 |
| **Temperate_WA** | **Summer** | 50 | 50 |
| **Temperate_WA** | **Autumn** | 0 | 100 |
| **Temperate_WA** | **Winter** | 50 | 50 |

This table was populated with the aid of empirical data obtained by [57], which shows that waterbird reproduction in the Kimberley region of WA (tropical) occurs mainly during summer, while in southwest WA (temperate) it occurs mainly in spring. In arid regions, seasonality *per se* was assumed not to affect the timing of the breeding season.

**Table S14:** The CPT of **Migratory_Species.**

| **Season** | **Migratory_Species** |
| --- | --- |
| **Spring** | Arrival |
| **Summer** | Arrival |
| **Autumn** | Departure |
| **Winter** | Departure |

In Australia, waterbird movement is more unpredictable than breeding season, but is usually oriented towards sites where water and food are available. Migratory wading bird species usually arrive at coastal and central regions of Australia in spring, before returning to the northern hemisphere in autumn [43]. Therefore waterbird populations are generally higher in spring and summer than in autumn and winter.

**Table S15:** The CPT of **Likelihood_of_Seasonal_Rain**.

| **Climatic_Zone** | **Season** | **Likelihood_of_Seasonal_Rain** | |
| --- | --- | --- | --- |
|  |  | **Yes** | **No** |
| **Tropical_WA** | **Spring** | 50 | 50 |
| **Tropical_WA** | **Summer** | 100 | 0 |
| **Tropical_WA** | **Autumn** | 50 | 50 |
| **Tropical_WA** | **Winter** | 0 | 100 |
| **Arid_WA** | **Spring** | 50 | 50 |
| **Arid_WA** | **Summer** | 50 | 50 |
| **Arid_WA** | **Autumn** | 50 | 50 |
| **Arid_WA** | **Winter** | 50 | 50 |
| **Temperate_WA** | **Spring** | 50 | 50 |
| **Temperate_WA** | **Summer** | 0 | 100 |
| **Temperate_WA** | **Autumn** | 50 | 50 |
| **Temperate_WA** | **Winter** | 100 | 0 |

This table was constructed based on the observation that the wet season of tropical WA occurs during summer, while that for temperate WA occurs in winter [27]. In arid parts of WA, rainfall does not closely follow a predictable seasonal pattern.

**Effects of the human community’s immunity to MVEV on risk (Part D)**

**Table S16:** Points of parent node states for **Immunity_Level_Popn** CPT.

| **Demographic_Structure** | | **Popn_Mobility** | | **MVEV_Epidemic_Previous_Year** | |
| --- | --- | --- | --- | --- | --- |
| **Higher_Propn_Young** | 0 | **High** | 0 | **Yes** | 1 |
| **Medium_Propn_Young** | 1 | **Medium** | 1 | **No** | 0 |
| **Lower_Propn_Young** | 2 | **Low** | 2 |  |  |

This table was constructed based on the rationale that younger individuals have less immunity to MVEV infection due to having lesser concentrations of protective antibodies [58]. Higher percentages of new immigrants (represented by the node **Popn_Mobility**) translate to greater risk to the community experiencing an outbreak because these individuals are expected to have lower levels of immunity to the virus (assuming that they had arrived from non-endemic areas). An epidemic in the previous year is expected to lead to greater prevalence of MVEV antibodies in the community, thus lowering the general risk of contracting MVEV disease in the present year.

**Factors directly influencing MVEV risk (Part E)**

In the following table, the maximum state of **Cx_annulirostris_Popn** was restricted by the state of **Mosquito_Habitat_Grid** (**Table S17a**). For example, if the state of the parent node is **Very_Good**, then **Cx_annulirsotris_Popn** can take all 5 states, but if the parent node state is **Good**, then **Cx_annulirostris_Popn** can take all states except **Very_High**; etc. The influence of all other parent nodes was weighted using the ‘points system’ as before (**Table S17b**). Restrictions were also placed on a number of other nodes such as **Ciconiiformes_Popn** and **Vector_Min_Infection_Rate**.

**Table S17a:** Restriction of **Cx_annulirostris_Popn** states based on the state of **Mosquito_Habitat_Grid.** A tick (√) means that state of **Cx_annulirostris_Popn** is permissible.

| **Mosquito_Habitat_Grid** | **Cx_annulirostris_Popn** | | | | |
| --- | --- | --- | --- | --- | --- |
|  | **Very_High** | **High** | **Medium** | **Low** | **Very_Low** |
| **Very_Good** | √ | √ | √ | √ | √ |
| **Good** |  | √ | √ | √ | √ |
| **Medium** |  |  | √ | √ | √ |
| **Poor** |  |  |  | √ | √ |
| **Very_Poor** |  |  |  |  | √ |

**Table S17b:** Points of parent node states for **Cx_annulirostris_Popn** CPT, except **Mosquito_Habitat_Grid**.

| **Past_3mo_Rainfall** | | **Popn_Growth_Rate_Combined** | | **Surface_Runoff_Seasonal** | |
| --- | --- | --- | --- | --- | --- |
| **Below_50mm** | 7 | **Ideal** | 4 | **Zero** | 1.5 |
| **From_50mm_to_100mm** | 10.5 | **Positive** | 3.5 | **Above_Zero** | 2 |
| **Above_100mm** | 14 | **Neutral** | 3 |  |  |
|  |  | **Negative** | * |  |  |
| * **Cx_annulirostris_Popn** is 100% **Very_Low** regardless of the state of any other parent node. | | | | | |

**Past_3mo_Rainfall** was judged to be more influential than the two other parent nodes, because rainfall is strongly associated with MVEV outbreaks (and *Cx. annulirostris* populations) in Australia [12, 29, 30]. **Surface_Runoff_Seasonal** has the smallest influence as it is theoretically correlated with **Past_3mo_Rainfall**, hence we did not want to ‘double-count’ the effects of both nodes. It was included to ‘compensate’ for areas where rainfall is low.

**Table S18a:** Restriction of **Ciconiiformes_Popn** states based on the state of **Bird_Habitat_Grid.**

| **Bird_Habitat_Grid** | **Ciconiiformes_Popn** | | | | |
| --- | --- | --- | --- | --- | --- |
|  | **Very_High** | **High** | **Medium** | **Low** | **Very_Low** |
| **Very_Good** | √ | √ | √ | √ | √ |
| **Good** |  | √ | √ | √ | √ |
| **Medium** |  |  | √ | √ | √ |
| **Poor** |  |  |  | √ | √ |
| **Very_Poor** |  |  |  |  | √ |

**Table S18b:** Points of parent node states for **Ciconiiformes_Popn** CPT, except **Bird_Habitat_Grid**.

| **Breeding_Season** | | **Migratory_Species** | | **Surface_Runoff_Seasonal** | |
| --- | --- | --- | --- | --- | --- |
| **Yes** | 10 | **Arrival** | 9 | **Zero** | 0 |
| **No** | 9 | **Departure** | 8 | **Above_Zero** | 1 |

The nodes **Breeding_Season**, **Migratory_Species** and **Surface_Runoff_Seasonal** do not have large influences on **Ciconiiformes_Popn** (signified by the small numerical difference between the weights assigned to the two states of each node), because (i) **Breeding_Season** and **Migratory_Species** can only capture general qualitative variations in breeding and migration of *Ciconiiformes*, and (ii) while large volumes of surface runoff are generally conducive to *Ciconiiformes*, the positive effects of water availability must be matched by the habitat suitability of an area. A similar argument can also be made for the low influence of **Surface_Runoff_Seasonal** on **Cx_annulirostris_Popn**. The combined influence of these three nodes is secondary to the influence of **Bird_Habitat_Grid**.

**Table S19a:** Restriction of **Susceptible_Ciconiiformes** states based on the state of **Ciconiiformes_Popn.**

| **Ciconiiformes_Popn** | **Susceptible_Ciconiiformes** | | | | |
| --- | --- | --- | --- | --- | --- |
|  | **Very_High** | **High** | **Medium** | **Low** | **Very_Low** |
| **Very_High** | √ | √ | √ | √ | √ |
| **High** |  | √ | √ | √ | √ |
| **Medium** |  |  | √ | √ | √ |
| **Low** |  |  |  | √ | √ |
| **Very_Low** |  |  |  |  | √ |

**Table S19b:** Points of parent node states for **Susceptible_Ciconiiformes** CPT, except **Ciconiiformes_Popn**.

| **Ciconiiformes_MVEV_Immunity** | |
| --- | --- |
| **High** | 8 |
| **Medium** | 9 |
| **Low** | 10 |

We set the influence of **Ciconiiformes_MVEV_Immunity** very low, because the node can only capture very general information about the immune status of *Ciconiiformes* based on the presence/absence of an MVEV epidemic in the previous year.

**Table S20:** The CPT of **Ciconiiformes_MVEV_Immunity**.

| **MVEV_Epidemic_Previous_Year** | **Ciconiiformes_MVEV_Immunity** | | |
| --- | --- | --- | --- |
|  | **High** | **Medium** | **Low** |
| **Yes** | 50 | 50 | 0 |
| **No** | 0 | 50 | 50 |

This node models the immune status of *Ciconiiformes* towards MVEV based on whether an MVEV epidemic had occurred in the previous year.

**Table S21a:** Restriction of **Vector_Min_Infection_Rate** states based on the state of **Cx_annulirostris_Popn.**

| **Cx_annulirostris_Popn** | **Vector_Min_Infection_Rate** | | | | |
| --- | --- | --- | --- | --- | --- |
|  | **Very_High** | **High** | **Medium** | **Low** | **Very_Low** |
| **Very_High** | √ | √ | √ | √ | √ |
| **High** |  | √ | √ | √ | √ |
| **Medium** |  |  | √ | √ | √ |
| **Low** |  |  |  | √ | √ |
| **Very_Low** |  |  |  |  | √ |

**Table S21b:** Points of parent node states for **Vector_Min_Infection_Rate** CPT, except **Cx_annulirostris_Popn**.

| **MVEV_Susceptible_Ciconiiformes** | | **WA_Region** | | **F_Adult_Lifespan_Combined (†)** | |
| --- | --- | --- | --- | --- | --- |
| **Very_High** | 16 | **Kimberley** | 16 | **Very_Long** | 8 |
| **High** | 14 | **Pilbara** | 12 | **Long** | 7 |
| **Medium** | 12 | **Rest_of_WA** | 8 | **Average** | 6 |
| **Low** | 10 |  |  | **Short** | 5 |
| **Very_Low** | 8 |  |  | **Very_Short** | 4 |
|  |  |  |  | **Unsuitable_Conditions** | * |
| ***Vector_Min_Infection_Rate** = 100% **Very_Low**, regardless of other parent node states. | | | | | |

†Minimum infection rates are dependent on the longevity of adult females as older females are more likely to be greater exposed to MVEV than younger ones. Longer-lived females are hence more likely to be infected with the virus (have a larger MIR) [Lindsay, pers. comm.].

**Table S22a:** Restriction of **Transmitting_Cx_annulirostris** states based on the state of **Vector_Min_Infection_Rate.**

| **Vector_Min_**  **Infection_Rate** | **Transmitting_Cx_annulirostris** | | | | |
| --- | --- | --- | --- | --- | --- |
|  | **Very_High** | **High** | **Medium** | **Low** | **Very_Low** |
| **Very_High** | √ | √ | √ | √ | √ |
| **High** |  | √ | √ | √ | √ |
| **Medium** |  |  | √ | √ | √ |
| **Low** |  |  |  | √ | √ |
| **Very_Low** |  |  |  |  | √ |

**Table S22b:** Points of parent node states for **Transmitting_Cx_annulirostris** CPT, except **Vector_Min_Infection_Rate**.

| **F_Adult_Lifespan_Combined (†)** | | **Extrinsic_Incubation_Period** | |
| --- | --- | --- | --- |
| **Very_Long** | 10 | **From_0_to_5_days** | 10 |
| **Long** | 9 | **From_5_to_10_days** | 9 |
| **Average** | 8 | **From_10_to_15_days** | 8 |
| **Short** | 7 | **From_15_to_20_days** | 7 |
| **Very_Short** | 6 | **Unsuitable_Temp** | * |
| **Unsuitable_Conditions** | * |  |  |
| ***Transmitting_Cx_annulirostris** = 100% **Very_Low**, regardless of other parent nodes. | | | |

†Longer-lived females increase the number of MVEV-transmitting vectors as they can transmit the virus multiple times [Lindsay, pers. comm.].

**Table S23a:** Restriction of **MVEV_Risk_Humans** states based on the state of **Transmitting_Cx_annulirostris.**

| **Transmitting_Cx_annulirostris** | **MVEV_Risk_Humans** | | |
| --- | --- | --- | --- |
|  | **High** | **Medium** | **Low** |
| **Very_High** | √ | √ | √ |
| **High** | √ | √ | √ |
| **Medium** | √ | √ | √ |
| **Low** |  | √ | √ |
| **Very_Low** |  |  | √ |

**Table S23b:** Points of parent node states for **MVEV_Risk_Humans** CPT, except **Transmitting_Cx_annulirostris**.

| **Nearest_Wetland_or_River** | | **Immunity_Level_Popn** | |
| --- | --- | --- | --- |
| **Below_15km** | 8 | **High** | 2 |
| **Equal_or_Above_15km** | 4 | **Medium** | 3 |
|  | | **Low** | 4 |

A maximum flight range of less than 15km was assumed for *Cx. annulirostris* [25]. **Nearest_Wetland_or_River**, a node which represents an objective quantity, was assigned a higher influence on MVEV risk than **Immunity_Level_Popn**, which represents, very generally, the immune status of human communities.
